# Supplementary material for: Are carbon emissions trading and green financial instruments synergistic? -Comprehensive quantitative research based on content analysis
Source: PLoS One. 2024 Mar 7;19(3):e0298601. doi: 10.1371/journal.pone.0298601 (PMC10919601; doi:10.1371/journal.pone.0298601)
Supplement: S1 Data — (ZIP) [file pone.0298601.s001.zip › Data/地级市Z_F工作报告.zip!/║┌┴·╜¡/╞▀╠¿║╙╩╨/└┤╫╘╣1⁄2╓┌║┼í╛┬φ┐╦╩2╛▌═°í┐_2012.docx]

各位代表：

　　现在，我代表市人民政府向大会作工作报告，请予审议，并请列席会议的政协委员和同志提出意见。

　　一、过去五年和2011年工作简要回顾

　　过去的五年，是我市发展史上不平凡的五年。面对国际金融危机的严重冲击，面对保增长、保民生、保稳定的繁重任务，在省委、省政府和市委的坚强领导下，在市人大、市政协的监督支持下，深入贯彻落实科学发展观，全力推进各项工作落实，圆满完成各项经济社会发展目标。

　　——综合实力明显提高。全市生产总值、规模以上工业增加值、财政一般预算收入分别是2006年的2.1倍、3.1倍、2.8倍，年均增长16.2%、25.6%、22.8%，增幅均位居全省前列。

　　——产业优势明显强化。形成煤-焦-化、煤-电-建材两条煤炭循环经济产业链条，焦炭产能达到1000万吨，占全省的67%；焦油加工产能15万吨，占全省的70%；焦炉煤气制甲醇产能28万吨，占全省的82%；粗苯精制技术填补全省空白，产能达到10万吨；煤化工产品达到21种，成为全省煤化工产业排头兵，区域竞争优势初步形成。

　　——城市形象明显提升。国家园林城市创建和“十项整治”活动取得实效，新增林地面积17万亩、绿地面积505万平方米，新建改造公园17处、景观带41处；新建改造城市道路26条、供水管线37.5公里、排水管线20.4公里，新增供热能力500万平方米、管道煤气用户4万户；取缔严重污染环境、浪费资源的企业10户，实施重点污染治理减排项目45个，是城市建设投资最大、项目最多、变化最显著的时期。

　　——发展活力明显增强。统一城乡八项政策标准，城乡一体化改革全省领先；调整市区行政区划，发展空间进一步扩大；经济开发区晋升为省级园区，成为全省财源建设示范园区；规范公务员及事业单位津贴补贴，财政供养人员工资水平居全省前列；政务服务中心建成运行，行政效率大幅提升；争取到国家循环经济、资源枯竭型城市经济转型、公立医院改革三个试点市，是得到国家政策支持最多的时期；非公经济快速发展，占全市经济比重提高3.6个百分点；成功举办四届七台河家具节，对外影响力显著提升；外贸进出口总额是2006年的8.4倍。

　　——人民生活明显改善。有入住要求的农村五保户和城镇“三无”人员全部实现集中供养，“五险”参保范围进一步扩大，社保标准进一步提高，教育、卫生等社会事业全面进步，高标准完成采煤沉陷区综合治理工程，“两棚一草”改造扎实推进，廉租房、经济适用房、公租房超额完成省政府下达任务。城镇居民人均可支配收入、农民人均纯收入比2006年分别增长85%、122%，年均增幅达到13.1%、17.4%。

　　2011年，是我市开创“十二五”良好开局的一年。一年来，市政府积极应对国内外宏观经济环境深刻变化带来的严峻挑战，全力上项目、保增长、惠民生，经济社会实现平稳较快发展。初步测算全市生产总值实现309亿元，规模以上工业增加值160.4亿元，固定资产投资120.6亿元，财政一般预算收入20.4亿元，扣除不可比因素分别增长10.1%、16.5%、35.6%、15.2%。外贸进出口总额实现9268.2万美元，增长50.2%。社会消费品零售总额63.6亿元，增长17%。

　　（一）凝神聚力抓项目，产业结构进一步优化。产业项目建设攻坚战成效显著，推进千万元以上产业项目82个，总投资99亿元，当年完成投资43亿元，其中，竣工项目51个,超亿元项目15个。煤化工产业提档升级。隆鹏98万吨焦炭、七煤72万吨焦炉改造项目投产，宝泰隆30万吨焦油加工和5万吨针状焦、龙盛达30万吨焦油加工、隆鹏12万吨甲醇、金泰6万吨环己酮和5万吨己二酸等项目开工。战略性新兴产业实现突破。成功引进德彩光电6万平方米LED显示屏项目，高新技术产业发展迈出新步伐。彪骐1.6万吨国际环保新材料项目投产，旺族太阳能光伏、巨达锂离子电池等项目加快推进，亚威200万平方米新型建材等9个产业项目落户经济开发区。招商引资力度加大。抽调优秀处级后备干部，组成33个专业招商小分队，离职两年驻外专职招商。组织企业参加哈洽会、港澳活动周等展洽活动，成功举办第四届七台河家具节。对上争取成效明显。强化对上争取意识，健全奖惩机制，加强督办检查，全年共争取各类项目200个，资金20亿元，增长35.1%。产业基础设施日趋完善。依七高速、七勃公路建成通车，勃利至林口铁路改造扩能工程完工，新民220千伏等9个输变电工程竣工，汪清水库、九龙水库等项目加快推进。建成七台河海关监管场所，结束我市无报关服务历史。9个产业园区初步形成，承载产业项目能力进一步增强。

　　（二）固本培基抓“三农”，城乡差距进一步缩小。粮食产量创历史新高。新建改造水利工程21处，新增大型农机合作社4个，土地承包经营权流转面积达到101万亩，规模经营面积达到68万亩，粮食生产能力显著提高，总产达到15.2亿斤。畜牧业发展提档加速。抢抓畜产品价高利好机遇，加快推进畜牧养殖规模化、标准化、科技化发展，新增标准化养殖场18个，拉动畜牧业规模化生产比重提高4个百分点。产业化水平整体提升。新建大兴牧业等4个大型产业化项目，新增省级龙头企业5户，大森林公司成为东北地区最大的肉鸡养殖加工企业。市级以上龙头企业实际加工能力超过90万吨，增长40%；实现销售收入25亿元，增长47%。28个农民专业合作社晋升为省级示范社。新农村建设扎实推进。投资2.6亿元加速推进城乡一体化，完成3个村整体搬迁，实施4个村整体改造，新建扩建乡镇寄宿制学校2所、标准化卫生院（所）61个、文化广场21个、通村公路168.9公里，解决35个村屯、3.7万人饮水安全问题。完成小城镇规划编制，2个省级百镇建设工程试点和5个市级小城镇建设试点加快推进，新建农民公寓楼24栋、9万平方米。

　　（三）突出重点抓改革，体制机制进一步创新。改革城投公司体制机制，注入83.6亿元有效资产，争取到开发银行近百亿元信贷额度，15亿元棚户区改造贷款落实到位，15亿元保障性住房企业债券发行有序推进。筹资2.3亿元组建东方博达产业投资公司，成为全省第一个地市级综合性股权投资平台。宝泰隆成功上市，结束我市无上市企业历史。与省科技厅签订科技合作共商协定，与哈工大等院校结为战略合作伙伴，建立哈理工国家大学科技园七台河分园。改革城建项目招投标体制，推行财政投资评审制度。集体林权制度改革全面完成。

　　（四）全民参与抓整治，城市功能进一步完善。坚持以加速城市化进程为突破口，完善城乡基础设施，改善城乡人居环境，提高城乡服务水平，累计投资23.3亿元，实施城建项目98个。国家园林城市创建扎实推进。高标准实施北出口景观改造升级等重点工程，完成30处绿化景观带、10处公园和广场绿化景观、15处节点绿化景观和5处林荫停车场建设，栽植树木109万株，植树造林2.4万亩，新增绿地面积50万平方米，受到国家园林城市验收专家组好评。“十项整治”活动深入开展。扩宽中心区主要街路11条，改造楼体立面110处，专项整治32个住宅小区环境，治理搬迁大型矸石山6处，亮化桃山、仙洞山、万寿山和大同街、学府街、山湖路。完成49项热网改造工程，新建供热管网8.3公里，维修改造供热管网26.7公里；实施9项供水改造工程，改造供水管网9.2公里，解决1.8万户居民吃水难题。兴凯湖引水工程完成规划批复。实施水源地保护工程4项、污染减排项目9项，第一污水处理厂投入运营。60户涉煤企业建成防尘除尘设施，9户焦化企业建成地面除尘站和生化废水治理站，重点控制污染企业环保达标水平提高12个百分点。完成主要街路信号灯岗、公交站亭、街道路牌和交通标志改造。调整出租车计价标准，完成出租车改色和计价器、LED灯安装，整顿出租客运市场秩序，服务质量明显提升。购进50台节能环保公交车，是政府公交投入最多的一年。城市管理长效机制初步形成。改革城市管理体制，整合城管职能组建新的城管局，积极推进“六化一考”管理机制试点，探索推行供热、绿化、道路、保洁等公用事业市场化运行体制。

　　（五）全力以赴抓民生，人民生活进一步改善。坚持财政资金优先向民生领域倾斜，七项民生工程、20件实事全面落实，城镇居民人均可支配收入实现16500元，增长10%；农民人均纯收入8241元，增长18.5%。安居工程加快推进。3个煤矿棚改续建小区竣工，北岸新城174万平方米煤矿棚改工程开工。开工建设14个城市棚改小区，当年竣工12个，安置居民1567户。新建廉租房2124套、公共租赁房1456套、经济适用住房200套，新增廉租住房租赁补贴家庭2715户。改造农村泥草房7254户、危房409户。社保能力稳步提高。启动城镇居民社会养老保险试点，新农保在全省率先实现全覆盖；上调企业离退休人员工资标准，月人均增资140元；失业保险实现市级统筹，支付标准月提高125元；城乡低保补助标准月提高28元，城乡低保人均财政补助标准月提高20元；廉租住房租赁补贴标准由月均60-100元上调至90-150元；城乡居民医保财政补助水平提高80元，人均基本公共卫生服务经费提高10元，新农合筹资标准提高80元，市内三级定点医保资金实现预付制和住院医保患者即结即报制，城乡医保住院报销比例提高5%。各项社会保障标准均位居全省前列。落实财政供养人员津贴补贴政策，首次发放住房提租补贴，月人均增资300元；住房公积金月人均增加72元；调整艰苦边远地区津贴补贴标准，月人均增加85元。特困小学生每年人均资助标准由160元提高到1000元，初中生由300元提高到1500元，高中生由1500元提高到2000元，并免交当年在校学习全部费用，实现特困生资助全覆盖。社会事业长足发展。累计实施校安工程项目68个、19.9万平方米，在全省率先完成建设任务。开工建设7所公办幼儿园，9所学校改扩建工程交付使用，建成全国首批百个残疾人艺术人才培训基地。国家公立医院改革迈出新步伐，“四个分开”改革全面启动，新出台19项惠民措施，勃利县中医院投入使用，完成4个社区卫生服务中心建设。城市无业人员计生奖励费用首次实现当年兑现，政府购买计生服务走在全省前列。市体育会展中心全面竣工，我市输送的运动员范可欣夺得2011年度短道速滑世锦赛500米冠军。群众性文化活动丰富多彩，乡镇文化站、村文化室、农家书屋在全省率先实现全覆盖。慈善事业快速发展，儿童福利中心和流浪儿童救助保护中心投入使用，红十字会救助病患困难户966户、重病患者102人，慈善总会“一日捐”活动资助贫困学生1000名。

　　（六）以人为本抓管理，和谐形势进一步巩固。以构建“八大工作体系”为重点，切实加强和创新社会管理。信访形势保持稳定。建立信访复查复核制度，完善信访三级终结机制，推行民情恳谈制度，设立信访维稳专项资金，开展领导大接访、“百日专项治理行动”、“信访积案化解活动”，越级访、集体访明显减少。社会治安形势良好。开展“警灯闪烁”等巡防活动，各类案件发案率大幅下降，破案率大幅提升，群众安全感普遍增强。安全工作全面强化。扎实推进“五化一加强”建设，初步完成煤矿周边水患排查，严格执行“12条高压线”制度，改革煤矿安全监管体制，组建煤矿安全隐患排查、执法稽查、巡视稽查三支队伍，成立煤矿安全考核办公室，实行监管人员轮岗交流制度，“两个主体责任”得到加强。应对突发事件能力明显增强，成功实施“8•23”透水事故抢险救援。道路交通、消防、危化、食品药品、特种设备等领域安全形势稳定，实现连续30年无重特大森林火灾。社区建设加快推进。建成10个城市示范社区和10个农村样板社区，服务居民能力进一步增强。

　　（七）转变职能抓服务，行政效率进一步提高。市政务服务中心全面推行重大项目“全程领办、特事特办、及时会办、联合办理、限时办结、跟踪督办”无障碍服务，群众满意率提高。全面启动“六五”普法活动，依法行政能力增强。严格落实市人大及其常委会的决议、决定，人大代表议案、意见、建议和政协委员提案办复率达到100%。政务公开、应急管理、政府门户网站建设水平提高。

　　过去一年，机构编制、统计、审计、外事、侨务、老龄、气象、地震、地方志、档案、人防、双拥、民兵、预备役等工作得到加强，民族宗教、新闻出版、妇女儿童等事业长足进步。

　　各位代表，这些成绩的取得，是省委、省政府正确领导、关心支持的结果，是市委统揽全局、科学决策的结果，是市人大、市政协有效监督、大力支持的结果，是全市上下团结奋斗、辛勤劳动的结果。在此，我代表市人民政府向各位代表和全市人民，向所有关心、支持七台河发展的各界人士，表示崇高敬意和衷心感谢！

　　在充分肯定成绩的同时，我们也清醒地认识到经济社会发展存在的突出问题。产业项目数量少、规模小、储备不足，难以支撑经济社会加快发展；产业结构单一，一煤独大局面没有根本改变，难以抵御煤炭市场波动冲击；现代服务产业起步晚、发展慢，土地、资金等生产要素相对紧缺，环境容量十分有限，支撑发展能力不足；各类安全生产事故暴露出的问题仍未得到根治，安全形势依然严峻；节能减排、生态建设和环境保护压力较大，基础设施、社会保障等民生投入有待于进一步提高。

　　二、未来五年发展目标和2012年政府工作任务

　　未来五年，是我市全面建设小康社会的攻坚时期，也是我市经济转型提速发展的关键时期。基于这一科学判断，刚刚闭幕的市八次党代会确定“加速经济崛起、建设幸福之城”为今后五年的奋斗目标，确定“建设经济繁荣的七台河、建设人民富庶的七台河、建设城市秀美的七台河、建设社会文明的七台河、建设充满活力的七台河”为今后五年的主要任务，确定“坚持以煤为基，着力推动四个发展；坚持项目立市，着力促进经济快速崛起；坚持改革创新，着力增强发展活力；坚持以人为本，着力提高群众幸福指数”为今后五年的工作原则，确定“到2016年，全市生产总值年均增长15%以上；财政一般预算收入年均增长16%以上；固定资产投资年均增长25%以上；城镇居民人均可支配收入年均增长14%；农民人均纯收入年均增长15%”为今后五年的主要预期指标。

　　“建设幸福之城”目标的提出，立足于我市经济向好发展的基本面，顺应了全市人民过上更好生活的新期待，是以人为本执政理念的深化和升华。“五个建设”任务的确定，抓住了科学发展这个主题和加快转变经济发展方式这条主线，完全符合科学发展观要求，符合七台河发展的历史阶段，是建设“幸福之城”的基本内涵和应有之义。“四个坚持”原则的明确，牢牢把握稳中求进的工作总基调，抓住了扩大内需这一战略基点、发展实体经济这一坚实基础、加快改革创新这一强大动力、保障和改善民生这一根本目的，突出了“稳增长、控物价、调结构、惠民生、抓改革、保稳定”的工作重点，符合国家经济政策导向，也符合市情实际。“主要预期指标”的提出，既充分考虑了广大人民群众盼发展、求富裕的强烈愿望，又统筹兼顾了我市现实的产业基础和未来的项目支撑，是经过反复论证，通过努力完全可以实现的指标。新一届政府将团结带领全市人民按照市委描绘的宏伟蓝图，全力抓好推进落实，力争早日建成繁荣富庶秀美文明的幸福七台河。

　　2012年是新一届政府履职尽责的开局之年，也是全市经济周期性调整的关键之年。一方面，经济社会发展面临诸多严峻挑战。世界经济复苏的不确定性、不稳定性因素增多，国际市场需求依然不足；国家稳定物价、抑制通胀政策趋向没有改变，企业扩张发展融资依然困难；全省产业项目建设加速，区域招商引资竞争更加激烈；煤焦市场低迷形势短期内难以改变，我市产业结构失衡造成的影响还会进一步加重，制约了经济周期性调整进程。另一方面，经济社会发展也面临难得机遇。国家着力实施扩大内需政策，重点加大保障和改善民生投入，信贷紧缩政策有所放宽，实施结构性减税政策，扶持战略性新兴产业发展；全省扶持产业项目力度加大，基础设施建设速度加快；我市业已形成的产业项目投融资、科技支撑等体制机制作用将逐步显现。所有这些积极因素，有利于我们积攒发展后劲、加速经济转型、保障改善民生、维护社会和谐。总的看，机遇大于挑战，只要我们把困难挑战估计得更充分一些，把应对措施安排得更周密一些，坚定信心、攻坚克难，科学应对、趋利避害，注重从变化的形势中捕捉和把握难得的发展机遇，扎扎实实把七台河的事情做好，就一定能走出一条调整时期加快发展的新路子。

　　2012年政府工作的总体要求是：以邓小平理论和“三个代表”重要思想为指导，深入贯彻落实科学发展观，按照市八次党代会战略部署，坚持以煤为基，安全发展，循环发展，转型发展，绿色发展，更加突出产业项目建设加快发展，更加突出统筹城乡发展促进“三农”，更加突出城市建设管理提升形象，更加突出实施惠民工程改善民生，更加突出社会管理创新保障和谐，全力推进经济、社会、生态、文化协调发展，向发展速度更快、运行效益更高、竞争实力更强、生态环境更美、人民生活更好的幸福之城目标迈进。主要预期目标是：全市生产总值确保增长12%、力争增长15%以上，财政一般预算收入增长16%以上，固定资产投资增长30%以上，城镇居民人均可支配收入增长12%以上，农民人均纯收入增长12%以上，万元GDP能耗下降4.2%。

　　（一）以强化产业项目建设为动力引擎，全力加快转变发展方式

　　强化产业项目建设，是七台河生存发展的根本所在，是七台河转型发展的前提基础，必须举全市之力坚决打好打胜这场攻坚战，推进实施超千万元产业项目90个以上，当年完成投资45亿元，其中，新开工项目50个。

　　一是进一步明确产业项目发展方向。立足推进煤化工等主导产业提档升级，放大国家循环经济试点市政策效应，按照《进一步促进煤化工产业健康发展的意见》，围绕以煤气为原料的合成化工体系、焦油为原料的精细化工体系、焦炭和焦油为原料的材料化工体系谋划储备项目。加快推进宝泰隆30万吨焦油加工和5万吨针状焦、龙盛达30万吨焦油加工、隆鹏12万吨甲醇、金泰6万吨环己酮和5万吨己二酸等项目，开工建设七煤90万吨向阳立井、铁东30万千瓦矸石电厂和隆鹏5万千瓦余热综合发电项目，谋划1.4亿立方米焦炉煤气制液化天然气、60万吨聚丙烯和4万吨尼龙项目。立足巩固发展传统非煤优势产业，用足用好国家资源枯竭型城市经济转型试点市政策，围绕木制品加工、机械制造、食品加工、中药加工等产业谋划储备项目。加快推进勃农兴达3万台(套)大马力拖拉机配套农机具、五方生猪养殖屠宰、兴盛石墨加工等项目建设，促进成石10万吨萘系高效减水剂等项目落地。立足抢抓国家重点扶持战略性新兴产业发展机遇，围绕新能源、新材料、节能环保、生物、信息、先进装备制造等产业研发储备项目。加快推进德彩光电6万平方米LED显示屏、巨达锂离子电池、旺族太阳能光伏、亚威200万平方米新型建材等重点项目建设，促进日拓9万台太阳能热水器、日久1万吨精细玻璃纤维、鹏程塑胶等项目开工。

　　二是进一步强化产业项目引进措施。突出强化专业招商上项目。进一步创新招商方式，强化招商措施，提升招商效果。强化专业招商考核，严格兑现奖惩政策，扩大专业招商影响力。突出承接产业转移上项目。抓住沿海发达地区产业升级机遇，强化与当地商会沟通联系，给足给优扶持政策，在承接产业整体转移上求突破。突出领导带头招商上项目。发挥各级领导对外联络广、交往层面高、协调能力强优势，带头外出招商，市政府领导和区县政府主要领导，两年必须引进1个单体投资5000万元以上的大项目。突出资源优势招商上项目。依托煤炭、风力等资源，积极引进勘探开发、矿井建设、风力发电等项目，杜绝资源占而不用行为，发挥资源招商最大效用。突出产业园区招商上项目。用好用活全省重点支持特色工业园区建设政策，重点推进以宝泰隆、隆鹏为核心的煤化工产业园区提档升级，力争建成全省煤化工产业核心园区、示范园区。以打造国家级经济开发区为目标，加快基础设施建设，强化招商引资和项目建设，全面提升经济开发区综合实力。整合升级县域产业园区，明确功能定位，理顺管理体制，创建省级勃利工业示范基地。谋划推进现代物流园区和互助精细化工园区。制定优惠政策，鼓励支持产业项目向园区集中。突出加强对上争取上项目。深入研究国家宏观政策，准确把握国家投资趋向，加强对上沟通联系，超前谋划包装对上争取项目，切实提高对上争取成功率，力争对上争取项目资金增长20%以上。

　　三是进一步优化产业项目建设环境。坚持把优化环境作为招商引资上项目的第一抓手，推行产业项目审批会办制，即时召集相关职能部门集中办理、限时办结，未按时办结的实施效能追责，全面提升行政审批效率。推行产业项目用地预留制度，统筹考虑，科学规划，超前收储足够的产业项目用地，确保项目引得来、落得下。发挥东方博达产业投资公司等融资平台作用，引导战略性新兴产业项目发展。支持企业上市融资、发行债券，解决产业项目建设资金不足难题。大力实施商标和名牌战略，引导支持企业争创著名商标、驰名商标和名牌产品，带动相关产业加快发展。

　　（二）以加快城乡一体化进程为主攻方向，全力破解“三农”发展难题

　　破解“三农”难题，是全面建设小康社会的难点所在，是实现城乡统筹发展的关键环节，必须以产业经济向农村发展，公共财政向农村倾斜，基础设施向农村延伸，城市文明向农村辐射，社会保障向农村覆盖为重点，着力缩小城乡差距。

　　一是加快发展现代农业。强化农业基础地位，突出发展特色种植、特色养殖、棚室蔬菜、劳务输出“四型”经济。全力扩大集约化、机械化、标准化、科技化种植面积，调整优化特色种植品种，确保粮食产能稳定在15亿斤。加快标准化蔬菜小区建设，棚室面积达到3万亩。积极争取大型农机合作社项目，实现大型农机作业能力全覆盖。积极推进土地承包经营权流转，规模经营面积提高20个百分点。加快推进中小河流治理、水库除险加固、灌区配套改造工程，切实增强抗御自然灾害能力。突出发挥龙头企业带动作用，促进畜牧养殖规模化、标准化、科技化、特色化发展，新增专业养殖场22个。加强劳动技能培训和就业信息服务，确保转移农村劳动力8.5万人，实现劳务收入6亿元。二是大力提升产业化程度。继续引进农副产品加工项目，引导现有龙头企业加强自主创新和技术改造，强化品牌创建、开发和整合，提升市场竞争能力。大力推进“企业+基地+农户”产业化模式，帮助龙头企业建立原料基地，新增农民专业合作社20个，带动形成种植基地150万亩以上。三是扎实推进小城镇建设。借鉴成都城乡一体化经验，完善小城镇建设规划，健全多元化投入机制，开发建设金沙新区和英伦小镇，深化2个省级百镇建设试点和5个市级小城镇建设试点工作，突出抓好撤屯并村工作，探索农民用宅基地置换城镇住房的有效途径，引导农民向城镇和新型农村社区集中。四是全面推进新农村建设。加强重点村、示范村试点建设，每个乡镇再打造一个重点村，完成宏伟镇富山村、铁山乡五星村、长兴乡东新村和中鲜村、永恒乡金山村、吉兴乡三民村6个整村改造项目。继续加快发展农村社会事业，加强农村基础设施建设，大力推进农村文化建设。

　　（三）以提升综合竞争能力为主要任务，全力加速推进城市化进程

　　加速城市化进程，是改善人居环境、完善城市功能的必然要求，是增强城市综合竞争实力的重要内容，必须按照市委建设绿色环保城市目标，扎实开展“三优”文明城市创建活动，着力建设环境优美、功能完善、秩序优良的七台河。

　　一是巩固提升国家园林城市创建成果。坚持把国家园林城市创建作为永不落幕的民生工程，启动三年绿化攻坚活动，实施石油大道绿化提档升级工程，改造提升城市公园、广场绿化景观，推进主要街路街景绿化、立体绿化升级，强化原有绿化成果和自然风貌管护，扩大临街单位、庭院、居住区、厂区矿区绿化战果，大力推行“绿色图章”制，提高城市绿化普及率和绿化景观档次。二是扎实推进基础设施建设。加快大同街东延工程及周边开发建设，完成石油大道东延和山湖路、东进街、大明街以及中心区次干道改造升级工程，加快老城区基础设施改造，抓好棚改腾空土地规划治理，推进七密、七宝等高速公路和鹤大公路七桦连结线改造前期工作，开工建设公路客运总站。实施勃利220千伏输变电工程等9个电力项目。完成汪清水库、九龙水库建设，加快推进桃山水库二期项目。启动亚行贷款供热管网改造及换热站建设工程。推进桃山水库坝下治理工程。完成勃利县污水处理厂、茄子河镇污水截流工程，实施市第二污水处理厂项目。三是全面深化“十项整治”活动。继续扩大整治范围，充实整治内容，提高整治标准，完善整治机制，巩固整治成果。突出抓好城区亮化、牌匾、空中拉线和厂区矿区整治。强化违法建筑监管，杜绝私搭乱建，坚决管住乱贴乱画等“十乱”行为。切实加强物业管理，全面改善住宅小区环境。实施市场化保洁新机制，解决环卫覆盖不彻底、垃圾清运不及时、保洁管理不到位问题。规范出租客运市场，调整优化公交网络，更新改造客运车辆，全面提升客运服务质量。严格落实污染物减排任务，强化空气污染治理，加快矸石山搬迁及生态恢复，取缔原煤散烧锅炉，继续推进涉煤企业防尘治理。推进倭肯河流域综合整治。四是健全完善城市管理长效机制。按照“六化一考”要求，研究出台标本兼治、重在治本、有效实用的城市管理办法和制度，重点整合公安、交警、城管数字平台，加快建设全市统一的数字监控系统，全面提升城市管理水平。五是着力提升城市服务功能。加快实施物流园区项目，全面完善物流服务设施。积极发展电子商务等新兴业态，大力引进咨询、评估、工程设计等商务服务机构，提升生产性服务业层次。改造传统服务业，加快发展旅游业，有序发展房地产业，大力发展文体娱乐业，优化发展餐饮业，突出发展社区服务业。

　　（四）以深化改革扩大开放为强大动力，全力推进体制机制创新

　　深化改革、扩大开放，是推动各项事业加快发展的不竭动力，是破解经济社会发展难题的有效措施，必须始终坚持这一基本点，积极构建充满活力、富有效率、更加开放的体制机制，以改革增活力，以开放促发展。

　　一是扎实推进重点领域改革。推进经济开发区和金沙新区管理体制机制改革，最大限度赋予区级行政职能，激发自主发展活力。推进强镇扩权改革，赋予试点镇部分区县职能，增强镇域经济自主权和发展动力。推进街道社区体制改革，开展区县直管社区试点。推进城市管理体制改革，组建城市管理委员会，下设管理指挥中心，理顺市区行政执法职能，建立城管、公安、工商、环保等部门联动执法机制。推进市政公用事业管理体制改革，建立公共财政补贴补偿机制，加快供水、供热、公交、环卫、园林等市场化进程。推进工程招投标体制改革，强化专家库、项目库、造价信息库建设，建立开标、评标、定标三分离机制。深化行政管理体制改革。推进事业单位分类改革。调整完善市区分税制政策。完善地方煤矿产量计量系统，强化涉煤企业税收监管。加强国有资产管理。推进城投公司严格按照现代企业制度运行，突出加强土地收储经营。二是全力做强做大非公经济。积极落实民间投资办企业优惠政策，最大限度地放宽准入门槛、降低注册标准、减免行政收费，积极帮助创业群众解决项目、用地、技术等难题。坚持外商内商一视同仁、内资外资一样优惠，支持本地企业和民间资本就地投资上项目。继续大力实施中小企业成长工程，鼓励引导企业创新技术、开发产品、扩大规模、打造品牌，走集群化、科技化、多元化发展之路，实现快速扩张发展。三是积极推进金融服务创新。完善政银企合作机制，政府出台重大经济政策、引进重大产业项目，邀请金融机构全程参与，做到信息共享共用，推进银企互信合作。支持金融机构抢抓国家放宽小型微型企业贷款条件机遇，结合实际创新金融产品，全力扶持小型微型企业发展。引导企业建立现代企业制度，规范财务管理，提高信用等级，争取银行支持。加快培育资本市场，规范民间借贷行为，盘活社会闲置资金，积极引进民办银行、证券机构、投资公司。四是全面提升对外开放水平。完成荣盛达国际物流中心建设，充分发挥海关监管场所作用，全面提升通关服务质量，出台优惠政策吸引周边外贸企业在我市注册报关，支持我市外贸主体扩大对外贸易，力争外贸进出口总额增长15%。支持企业走出去参加各类展会，赢得更多合作发展机会。办好第五届七台河家具节，筹办短道速滑邀请赛等体育赛事。推进对外文化交流，增进国际友好城市往来。

　　（五）以提高群众生活水平为根本目的，全力改善和保障民生

　　改善和保障民生，是经济社会加快发展的根本目的，是地方政府履职尽责的第一目标，必须集中财力，推进落实一批民生实事，稳步提高人民生活水平。

　　一是加快安居工程建设。坚持把保障性安居工程建设作为解决中低收入家庭住房困难，遏制房价过快上涨的首要措施。（1）完成北岸新城煤矿棚改续建工程，安置棚户区居民2.5万户；开工建设剩余煤矿棚改工程。（2）新开工城市棚改小区14个，回迁居民3069户。（3）新建廉租住房4600套、公共租赁住房300套、经济适用住房333套，新增廉租住房租赁补贴家庭2500户。（4）足额建设农村因灾倒房，改造农村泥草房5000户。二是严格兑现社保政策。（5）城市低保补助标准由每月258元提高到274元，城市低保人均财政补助水平由每月181元提高到197元。（6）城乡居民养老保险参保率达到70%，继续做好被征地农民养老保险工作。（7）上调企业离退休人员工资。（8）继续扩大基本药物制度实施范围,城乡参保居民住院报销比例由65%提高到70%以上。（9）推广市、县就医“一卡通”，实现参保人员统筹区域内医疗费用即时结算。三是突出发展社会事业。（10）实施教育优先发展战略。创新机制推进公办幼儿园建设，完成7所幼儿园续建工程；加强标准化学校和优质高中建设，扩建2所学校，完成4所学校续建工程；大力发展职业教育，促进学前、义务、高中、职业教育均衡发展。调整优化学校布局，推进乡镇集中办学，改善城乡学校食宿条件，缩小城乡、区域办学差距。（11）完善城乡公共卫生服务体系。深化医药卫生体制改革，扎实有效推进国家公立医院改革试点，整合资源异地新建市中医院、市妇幼保健院、市精神病医院，新建市人民医院外科楼、勃利县人民医院儿内科楼，新建改造6个社区卫生服务中心和55个乡镇（村）卫生院（所）。完成食品药品检测中心建设。（12）大力促进文化发展。深入总结挖掘七台河精神，增强全市人民的归属感、认同感、自豪感。深化文化体制改革，增强文化事业发展活力。健全公共文化服务体系，谋划推进市文化中心、广电中心、图书馆、煤炭博物馆、烈士陵园项目，完善基层文化基础设施。深入开展“七台河之夏”等品牌文化活动，丰富城乡群众文化生活。推进文化产业发展，支持勃利黑陶、森帝酒具、松林玉木雕等产业基地建设。培育现代文化市场体系，组建市级文化产品营销市场。设立文化发展基金，保障重点文化项目、文化精品生产、文化人才培养等资金需求。深入开展全民健身运动，全面提升竞技体育水平。发挥体育名人效应，推进体育产业发展。（13）加快发展残疾人事业。开工建设残疾人康复中心，免费实施白内障复明手术120例，提供残疾人用品用具及辅助器具400件。（14）全面做好人口工作。切实稳定低生育水平，提高人口素质，统筹解决人口问题，继续推进“惠家”工程，建立健全计生利益导向机制，不断提高家庭发展能力。四是强化便民利民措施。（15）建立“民生110”公共服务中心，城管等职能部门和供热、供水等公用事业单位全员参与，实行24小时接听，统一受理，分级交办，及时督办，限时办结。（16）按照城市公厕人口分布需求新建一批公厕。五是千方百计扩大就业。（17）强化政策支持和资金扶持，加大就业困难人员就业援助，支持劳动者自主创业和自谋职业，培养“小老板”1000人，引导高校毕业生到城乡基层、中小企业就业和自主创业，发放小额担保贷款4000万元，城镇登记失业率控制在4.3%以内。六是做好稳价保供工作。（18）密切跟踪价格走势，严格价格调控机制，确保人民群众不因物价上涨影响生活质量。进一步畅通外地农副产品进入我市的绿色通道，严格执行农副产品储备制度，保证日常生活用品供应充足。七是实施饮水安全工程。（19）加快推进兴凯湖引水工程，力争开工建设。（20）强化水源地污染综合治理，建立水源地保护长效机制，加强倭肯河湿地保护区建设。

　　（六）以加强创新社会管理为有效途径，全力促进社会和谐稳定

　　加强和创新社会管理，是做好新时期群众工作的关键举措，是维护社会和谐稳定的主要抓手，必须按照市委推进“八大工作体系”建设要求，以“化解社会矛盾，满足社会需求”为重点，创新工作措施，切实解决好影响社会和谐稳定的突出问题。

　　一是全力做好安全生产工作。牢固树立安全为天理念，一时一刻也不放松地抓好安全生产工作。全面强化以煤矿为重点的安全生产工作，严格落实“五化一加强”措施，健全完善安全生产制度，强化从业人员培训，加强井下避险“六大系统”建设，全面提升煤矿安全生产条件，推进本质安全型矿井建设，促进企业安全生产主体责任落实。充分发挥煤矿安全隐患排查、执法稽查、巡视稽查三支队伍作用，加大监管人员轮岗交流力度，切实强化矿井日常安全监管，严格落实政府安全监管责任，确保煤矿不发生较大以上事故。突出食品药品安全监管，建立健全部门联动机制，严厉打击制售、贩卖假冒伪劣食品药品行为。严格危险化学品生产、储运、使用等环节管理，强化重点领域、关键部位防范措施，严防爆炸、泄漏等事故发生。全力抓好道路交通、消防、易燃易爆、非煤矿山、森林防火、特种设备等领域的安全生产工作。二是全面加强社会治安综合治理。深入开展“平安七台河”创建活动，强化社会治安人防、技防措施，切实加强网络舆情监管，提高预防和应对各类突发事件能力。始终保持对违法犯罪高压态势，严厉打击各类违法犯罪行为，有效遏制恶性案件发生。三是突出抓好信访稳定工作。落实落靠信访责任制，完善社会矛盾排查、决策风险评估、社会化大调处机制，坚持民情恳谈和领导接访下访制度，及时掌握社会舆情动态，加大积案化解力度，切实解决群众反映的突出问题。坚持依法治访，依法处理缠访、闹访、无理访，全力维护社会和谐稳定。

　　三、全面加强政府自身建设

　　建设人民满意政府是一项长期而紧迫的任务，新一届政府必须按照“尽职责、强作风，求实干、促发展，重学习、提素质，讲团结、增合力，抓廉政、树形象”要求，以良好的精神状态、创新的发展思路、科学的行政方式、务实的工作作风，开创各项工作新局面。

　　一是努力建设学习型政府。着眼于提高思想政治水平和行政履职能力，加强党的路线、方针、政策特别是科学发展观理论的学习，突出掌握现代市场经济、社会管理、科技、金融、法律等方面知识，坚持理论联系实际，学以致用、用以促学，不断提高谋划发展、统筹发展、优化发展、推动发展的本领，提高驾驭复杂局面和应对突发事件的本领，提高做好群众工作、维护社会稳定的本领。

二是努力建设服务型政府。深入推进行政审批制度改革，加强政务中心建设，推行服务质量承诺制，提高社会管理和公共服务水平，为各类市场主体创造良好发展环境。深入开展“农事村办、企事厂办、居民事社区办”、“公共服务和生活环境细节最佳最差评比”、“十进社区”、“联企、包区、助村、帮户”等活动，推动政府职能部门、司法机关、公共服务单位重心下移，为群众提供更加便利的服务。

　　三是努力建设法治型政府。自觉接受人大依法监督，主动接受政协民主监督，及时办理人大代表议案、意见、建议和政协委员提案。扎实推进“六五”普法活动，全面加快依法治市进程。健全科学民主决策制度，完善重大事项公众参与、专家咨询论证和政府集体决策相结合的决策机制，提高决策科学化、民主化水平。深化行风评议，加强行政监察和审计，落实行政执法责任制和问责制，杜绝不作为、乱作为现象，严厉查处失职、渎职行为。

　　四是努力建设廉洁型政府。模范遵守廉洁从政准则，严格落实党风廉政建设责任制，强化重大决策部署落实情况监督检查，深入开展重点领域突出问题专项治理，严厉查处腐败案件，切实纠正损害群众利益的不正之风。

　　各位代表，新的一年孕育着新的希望，面对新的机遇、新的挑战，让我们在市委的正确领导下，深入贯彻落实科学发展观，以奋发有为的精神状态、开拓创新的进取精神、求真务实的工作作风，坚定信心，迎难而上，为早日把七台河建成幸福之城而努力奋斗！
